# External Application of Chloroform in Erysipelas

**Published:** 1851-09

**Authors:** A. L. Castleman


ARTICLE IV

### *External Application of Chloroform in Erysipelas.*

[For the following, communicated in a letter from Alfred L. Castleman, M. D., of Wis., we return our thanks, and hope our readers may be favored with frequent communications from his pen.]

I have this morning read in the N. W. Med. and Surg. Journal an article, accredited to the Ohio Med. and Sur. Journal, on the "*Accidental* employment of Chloroform in Erysipelas." As the cases cited here are too few to give the remedy much weight in the treatment of the disease, I wish to say that for some months I have used it (though not accidentally)

in the treatment of this disease, and in every instance with the most marked benefit. I at first used it combined with Olive Oil, in the proportion of three parts chloroform to one of the oil. I now, however, use it pure, unless in cases where there is much pain, when I add a little Sulp. Morphine.

I do not recommend the use of Chloroform to the profession as a remedy to be relied on in the treatment of Erysipelas. My experience in the use of it is yet too limited, and medical men cannot be too careful in bringing forward new remedies on *slight* evidence of their remedial power. I should not have made this communication but for having seen the one above referred to, and with the hope that it may call forth in your September issue any further evidence that can be adduced on this point.

*June* 21*st*, 1851.

